# Hypochlorous Acid Can Be the Novel Option for the Meibomian Gland Dysfunction Dry Eye through Ultrasonic Atomization

**DOI:** 10.1155/2022/8631038

**Published:** 2022-01-05

**Authors:** Zhiyuan Li, Haiyan Wang, Mo Liang, Zhenghua Li, Yvliang Li, Xiaoping Zhou, Guoping Kuang

**Affiliations:** ^1^The Diagnosis and Treatment Technology Research and Development Centre for Dry Eye and Ocular Surface Disease of Chenzhou, Chenzhou, 423000 Hunan, China; ^2^The Ophthalmology Department of the Affiliated Chenzhou Hospital, Hengyan Medical School, University of South China, Chenzhou, 423000 Hunan, China; ^3^The Operation Department of the Affiliated Chenzhou Hospital, Hengyan Medical School, University of South China, Chenzhou, 423000 Hunan, China

## Abstract

**Background:**

Dry eye is a multifactor disease which needs comprehensive treatments to keep the homeostasis of ocular surface.

**Objective:**

To explore the effect of hypochlorous acid on the meibomian gland dysfunction dry eye through ultrasonic atomization.

**Methods:**

We set this study of 0.01% HOCL and 0.1% hyaluronate by ultrasonic atomization. All the data was recorded at the 1^st^, 15^th^, 30^th^, and 55^th^ days. The patients' complains, the meibum analysis, conjunctive congestion, corneal staining, Schirmer's I test, and NIBUT were recorded by K5M, the MMP-9, and IL-2 of tear by inflammation kit; the *Demodex* was recorded by microscopy.

**Results:**

53 patients have joined this study. There is no statistic difference between them on OSDI (day 15: *p* = 0.061, 30: *p* = 0.055, 55: *p* = 0.052); results show the 10.57 ± 0.13 and 12.54 ± 0.17 reduction on OSDI; the differences of both treatments are significant (^∗∗^*p* < 0.01). Increased Schirmer's and TBUT are 3.27 ± 0.10 and 6.29 ± 0.10 (^∗∗^*p* < 0.01) or 7.32 ± 1.72 s and 9.22 ± 1.41 s (^∗^*p* < 0.05); the decreased conjunctive and corneal staining are 0.23 ± 0.07 and 0.45 ± 0.06 (^∗∗^*p* < 0.01) or 0.42 ± 0.03 and 0.37 ± 0.02 (^∗^*p* < 0.05) at both groups. The differences of MMP-9 and IL-2 negative rate are significant (*Z* = 0.896, ^∗∗^*p* = 0.002 < 0.01; *Z* = 0.659, ^∗∗^*p* = 0.001 < 0.01); the number of *Demodex* mites at first is 10 or 11, while the last is 2 or 6 (*Z* = −4.642, ^∗∗^*p* < 0.01; *Z* = 2.742, *p* > 0.05). The *Demodex* count between them is significant (*Z* = −2.310, ^∗^*p* = 0.032 < 0.05). The survival times (ST) of each stage at the HOCL are 110.75 (108.50 ± 24.50), 95.50 (90.25 ± 14.50), and 75.25 (73.48 ± 8.50) min which are shorter than those of control which are 155.50 (160.10 ± 21.50), 130.25 (128.25 ± 16.50), and 105.75 (102.50 ± 14.50) min (^∗∗^*p* < 0.01). The *Demodex* eradication rate of HOCL is statistic significant (^∗^*p*15^th^ vs. 1^st^day = 0.028 < 0.05; ^∗∗^*p*30^th^ vs. 1^st^day = 0.002 < 0.01; ^∗∗^*p*55^th^ vs. 1^st^day = 0.0018 < 0.01).

**Conclusions:**

0.01% HOCL improves the *Demodex* eradication by shortening the survival time; the HOCL acts on the ocular surface by reducing the inflammation. The ultrasonic atomization helps for the drug usage.

## 1. Introduction

The Dry Eye Work Shop (DEWS) II (2017) report has rewritten the dry eye disease (DED) definition: “Dry eye is a multifactorial ocular surface disease characterized by loss of tear film homeostasis associated ocular symptoms, in which tear film instability and hyperosmolarity, inflammation and ocular surface lesions, as well as neurosensory abnormalities play etiologic roles” [1]. The major prevalence globally ranges from 5–7% of the USA to 40–70% of the Eastern Asian [2]. The most vulnerable age of the disease is middle and elderly people (age > 40 years), but the incidence is rising among the young [3, 4]. It is crucial for the public to realize the importance of DED by reliable therapy.

Meibomian gland dysfunction (MGD) is defined as a team of aberrant signs, acquired or congenital, implicated by dysfunction of the eyelid meibomian glands. MGD leads to affecting tear film function, ocular discomfort, evaporative caused dry eye, or ocular surface problems [5]. The study of 619 randomly chosen participants from a population-based study in north China; 8.6% were symptomatic MGD while the asymptomatic MGD rate was 21.9% [6]. Many of these ocular clinical manifestations interweave with dry eye, and *Demodex* relative MGD is believed to be the key pathological factor to evaporative induced dry eye [7].

Kinds of means are used for the therapy of MGD-related DED, containing nondrug treatments such as Traditional Chinese Medicine (TCM), ultrasonic atomization (UA), intraductal eyelid meibomian gland probing, meibomian gland massage, optimized pulsed light therapy (OPT), and lipiflow. Consider these means are effect to a certain range; difficulties are the inappropriate usage, insignificant effects, high financial expenditure, and the secondary actions [8]. It is necessary for the invention of new proposal to improve the effect.

Hypochlorous acid (HOCL) has multifaceted applications in dermatology, wound healing, eye care, and dentistry. It is the ordinary disinfectant in industrial domestic and medical aspects and has the same active components of household bleach but with a different chemical composition [9]. HOCl is an attractive material for the nonsynthetic microbial toxic medium. Impurity-free HOCl originated from the products of the human immune response [10]. Across the oxidative reaction, highly activated molecules such as HOCl are stimulated from the leukocyte's action to external microorganisms [11]. Due to its quickly neutralized feature, HOCL is nontoxic to the ocular surface [12].

Ultrasonic atomization (UA) [13] is a procedure that damages liquid surface tension and atomizes drops into cute elements through ultrasonic vibration. It is the conventional ophthalmological choice in TCM for DED. This procedure of 20 min makes the droplets fully expose and permeate the ocular surface. Motivation of anatomized steam can accelerate body fluid circulation around eyelid and thus reinforce the release of meibomian gland secretions.

Anyway, concentrate on this earlier work to assess the particular therapeutic safety and effects of ultrasonic-atomizer HOCL for the remedy of MGD-related DED which is necessary. Until now, this is the premier double-blind, placebo-controlled randomized study for the hypothesis.

## 2. Participants and Evaluations

### 2.1. Study Design and Participants

A double-blind layout was designed in this clinical research. Thus, the 1 : 1 ratio of HOCL and placebo treatment groups was carried out to engender random codes using the statistics software SAS9.4 (SAS Institute Inc., Cary, NC, USA), under the block randomization modus. Observed were then followed a medication box labelled with a bunch of number which contained all medicaments. The clinical research group used market-oriented available HOCL 0.01% pH 5.4 (20 mL) (Avenova®, NovaBay Pharmaceuticals, Inc., Emeryville, California, USA) [14]. At the same time, the control was treated with a placebo material 0.1% Purified Sodium Hyaluronate (20 mL) (Santen Pharmaceutical Co. Ltd.) which is a 0.72% (g/mL) sodium chloride solution with the osmotic pressure of 235 mOsmol/kg and a pH value of 6.5. Patients were also treated with fixed bracket ultrasonic atomizer (product number: HL100A, Yuwell, Jiangsu, China). The medications in this study were atomized for 20 min per 5 days with the ultrasonic atomizer held before the patient's eye. All the patients were treated with meibomian gland massage once a week, 4 times altogether. The treatment duration was 55 days with check point time at the 1^st^ day (baseline) and then the 15^th^ day, 30^th^ day, and 55^th^ day; treatment safety and effectiveness evaluations were executed on-site during the follow-ups. All trails were done on the same instrument. The investigator and the statistician know nothing of patients' identity.

### 2.2. Inclusion and Exclusion Criteria

All the study subjects were enrolled during Jan 2020-June 2021 period who met the inclusion criteria and were of the same nationality (Han Chinese, aged from 20 to 70 years). Patients with MGD-DED were chosen from those subjects who visited the eye clinic for dry eye.

We excluded patients less than 20 or more than 70 years old; those who with the histories of ocular injury and surgery within 3 months; those with ocular problems such as ocular inflammation, allergy, and nasolacrimal sac problems; those who are using a punctual plug or contact lenses; or those are using eye drops including high-quality artificial tears drops within 24 hr before the examination. Anyone whose outcome results were hard to be confirmed was excluded.

### 2.3. Ethnic Achievement

This study followed the principium of the Declaration of Helsinki, and this protocol was approved by the institutional review board of the ethics committee of the Affiliated Chenzhou Hospital, Hengyan Medical School, University of South China (ID: 2020YJ03). Informed consent was achieved by the recruiters after a discussion of the purpose and probability on sequences of the clinic trail.

### 2.4. Dry Eye Diagnosis and Classification

- The standards for the general normal are as follows [15, 16]:

1. Ocular surface disease index (OSDI) score of less than 12
2. Without tear film outliers (tear film break-up time, TBUT > 5 seconds, and Schirmer's test value of >5 mm after 5 min)
3. Lack of evidence of corneal or conjunctive epithelial erosion with fluorescent staining
4. Normal lid margins or meibum

(ii) The criteria for the aqueous-deficient dry eye (ADDE) group are as follows [15, 16]:

1. Presence of dry eye complains (OSDI ≥ 12)
2. Poor tear production as defined by Schirmer's test I (≤5 mm) and tear film instability as named by the FBUT(≤5 seconds)
3. The evidence of corneal or conjunctivae epithelial damage with a fluorescent staining score of ≥3

(iii) The criteria for the MGD group are as follows [15, 16]:

1. Presence of symptoms (OSDI ≥ 12)
2. At least one lid margin abnormality
3. Poor meibomian gland expression (grade ≥ 1) or worse qualitative variety in meibum (meibum quality score ≥ 3).

(iv) The criteria for the ADDE/MGD-related dry eye group are as follows [15, 16]:

1. Presence of dry eye complaints (OSDI ≥ 12)
2. Poor tear production as recorded by Schirmer's test I (≤5 mm) and tear membrane film instability showed by the FBUT(≤5 seconds)
3. The evidence of corneal or conjunctivae epithelial discontinue with a fluorescent staining score of ≥3
4. Not only one lid margin abnormality
5. Worse meibomian gland expression (grade ≥ 1) or poor quality changes in meibum (meibum qualitative score ≥ 3).

### 2.5. Ultrasonic Atomization Process [17]

Patients were also assembled with fixed wing ultrasonic nebulizers (product Number: HL100A, Yuwell, Jiangsu, China). The atomizing pipeline was placed 5–10 cm before the eyelid, and the patients open their eyes larger and stare in all orientations off and on to ensure that the ultrasonic atomizing fine droplets fully penetrate the conjunctive. Treatment fluid was used 20 mL HOCL or placebo each time. The body temperature of an ultrasonic-atomized aerosol is very close to the room environment; make the entire process arousing the least irritations. The therapy period was 55 days with check points at time day one (baseline) and then at the 15^th^ day, 30^th^ day, and 55^th^ day; treatment safety and efficacy assessments were implemented on-site during these visits.

### 2.6. Clinic Assessment

One author of our team performed the experiments, and data were obtained from both eyes. In all the subjects, clinic results were taken sequentially as follows:

Subjective symptoms were graded on a serial scale from 0 to 4, according to the verified 12-item ocular surface disease index (OSDI) questionnaire. The total OSDI marks range from 0 to 100 and was calculated using the following equation: OSDI = (the summary of scores for every question answered × 100)/(overall number of answered questions × 4) [18].

The following are the objective data through Oculus K5M:

1. Tear meniscus height (TMH) was recorded by Keratograph® 5M (K5M; Oculus, Optikgerate, Germany). The keratograph was set to “film (TF) scan-tear meniscus mode” to capture the image of the TMH of the ocular surface, following the manufacturer's synopsis, as previously reported [19, 20]
2. Tear membrane film evaluation with the “TF-Scan, Non-Invasive Keratograph Break-Up Time (NI-KBUT) mode” was chosen when the subjects were asked to blink three to five times and then keep their eyes open. There is abnormality on the manifested destructive or break-up of the tear film; meanwhile, the picture was recorded. The equipment provided a representative of tear film break-up all the time, including a tear film topographic map showing the size and location of the tear discontinue regions, as well as the first break time (NIKBUT-first) and the average break-up time (NIKBUT-average; the meanings of all tear film break-up over the entire cornea), as previously described [21]
3. Conjunctive and corneal staining was graded from 0 to 3 and corresponds to the National Eye Institute (NEI)/Industry Workshop scale [20] from 0 to 33 based on the type of fluorescent staining under the slit lamp
4. Schirmer's examination I was performed without topical anaesthesia by placing Schirmer's strip in the1/3 middle lateral part of the lower fornix. The length of wetting was recorded after 5 min, and the patients were asked to make their eyes slightly closed during the test [22]
5. The eyelid margins and meibomian glands were examined for lid margin anomalous, gland expression, and meibum amount and colour, as previously described [23, 24]. Lid margin anomalous were scored as 0 (absent) or 1 (present) for the following parameters: plugged meibomian gland orifices, vascular congestion, irregularity of the lid margin, and partly expressions of the mucocutaneous borderline [25, 26]. The extent of meibomian gland expression, using steady digital pressure applied on five glands of the middle third of the lower lid, was graded as such: grade 0, all five glands expressible; grade 1, three or four glands expressible; grade 2, one or two glands expressible; and grade 3, no glands expressible [27]. The meibum attribute on eight lower lid glands was graded as follows: grade 0, clear; grade 1, cloudy; grade 2, cloudy with granular particulates; and grade 3, thick, like toothpaste-like particulates. Each of the eight glands of the lower eyelid was graded on the scale from 0 to 3. The scores of the eight glands were summarized (range: 0–24) [28]

The following are the data from optical microscopy for *Demodex*:

1. For *Demodex* count [29] on each eye, pluck out 3 upper and lower eyelashes. Try to select the eyelashes with sleeve-like scales at the roots. Clamp the eyelashes with tweezers, and rotate them slightly to loosen the eyelashes. After plucking, place them in parallel on the glass slide. Observe the amount and morphology of *Demodex* mites under the optical microscope. If the plucked eyelashes are accompanied by scales, add 100% alcohol or 0.25% fluorescent sodium solution and observe again. The *Demodex* count includes its life cycle
2. For the survival time (ST) of *Demodex*, written informed protocols have been acquired from each patient before cilia have been removed. After eyelashes have been pulled out from the participants' eyelid in each group at room temperature, these eyelashes with *Demodex* mites were quickly fastened on the glass slide. HOCL and placebo eyelid patch extracts were then layered onto the glass slide with a micropipette individually. The original sample was treated as the blank origin group. As *Demodex* is more vulnerable at the young stage of life, only adult *Demodex* with four pairs of well-developed legs and a robust body were found [30]. After the cilia had been extracted from the eyelid, the activity of the *Demodex* body and legs was observed instantly and continuously under the ordinary optical microscope at the magnification of ×10 or ×40. The ST was named as the period from the time-point of eyelid patch extract trickled on the body to the cessation of activity
3. In *Demodex* mite eradication, only the data of *Demodex* mites was ≥3; the patient was considered *Demodex*-positive [30]. Otherwise, “absolute *Demodex* eradication” was defined as complete *Demodex* eradication with the tissue reduced to zero [31]

### 2.7. Tear Film Protein Factors [32]

Tear specimens were obtained using a diagnostic test reagent strip in order to asses with inflammatory kits (Inflammation Dry® test; Rapid Pathogenesis Screening Inc., Sarasota, FL, USA) for each patient. Microbiological pieces were collected before and 20 minutes after implementation of the procedure.

### 2.8. Sample Size Calculation

There was not a previous study that has directly related studied the subjective complains and objective data with ocular eyelid ultrasonic atomization for disinfection. The arbitrary effect size of 1-*β*¼0.80 and 0.7 at *α*¼0.05 [33, 34] (a priori, two-tailed, matched-pair test) was selected to count the minimum sample for this study and was estimated to be 25.

### 2.9. Statistical Analyses

The main variables did not have a normal distribution; nonparametric tests were used. The clinical variables and ocular surface index were compared between the control and HOCL ultrasonic atomization using the Analysis of Variance (ANOVA) and for categorical variables using Fisher exact or chi-square test. A confrontation of clinical manifestations and ocular surface index is due to the presence of a simultaneous multiple tear membrane film break-up pattern and ocular surface index by the K5M; the observation index does not coincide with the normal distribution and is expressed as the median P50 (P25, P75). Statistical analyses were performed using SPSS (version 19.0; SPSS Inc., Chicago, IL, USA). A *p* values less than 0.05 was considered statistical significant.

## 3. Results and Analysis

### 3.1. The Clinic Trail Diagram for the Procedure for the Safety and Efficiency of HOCL and Placebo Drug Ultrasonic Atomization

**Figure 1:**
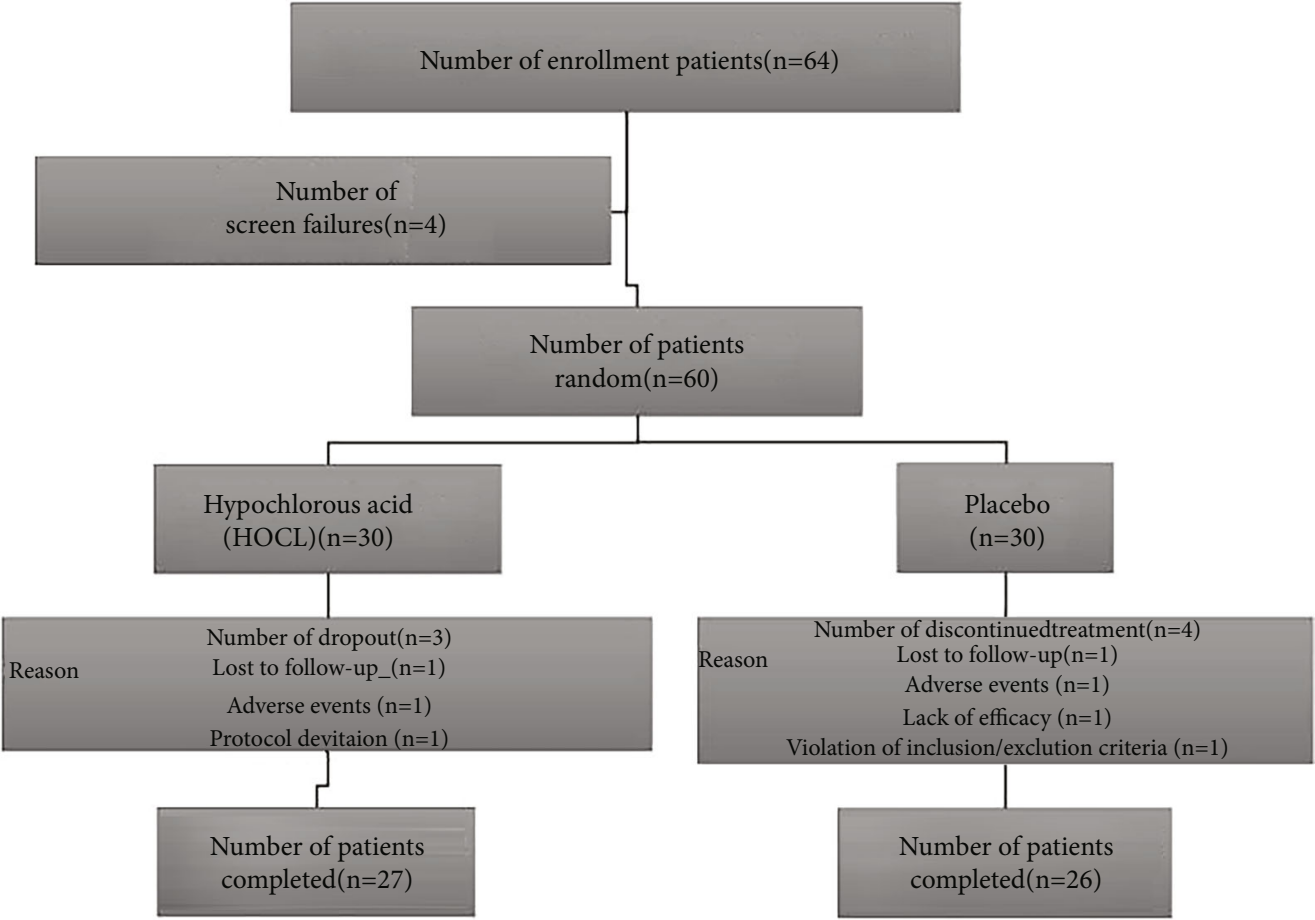
Subject flow chart.

These 64 recruited patients were randomly selected for double-blind treatments. Two persons in the HOCL and placebo group did not attend screen schedule; accordingly, 30 patients were analyzed in these groups separately. A total of 27 or 26 patients have finished all clinic visits, respectively; this shows that only 1 patient dropped out from each group for adverse events (transient conjunctive hyperaemia) (Figure 1).

### 3.2. Basic Clinical Information of Enrolled Patients

If the patient wore glasses, the best corrected visual acuity should be recorded, without recording the uncorrected visual acuity. If the patient wore no glasses, the uncorrected visual acuity was recorded. The data of subjects in the designated treatment and amount of subjects in the specified systematization of percentages were based on the number of subjects in the homeostasis treatment. The data in Table 1 show patient clinical idiographic at the beginning. These data show no differentiation in demographic distinctive between them. Results also show no statistic differences at baseline between them in signs of MGD and/or DED as well as in terms of ocular symptoms.

For persistent variables, *p* value was calculated using Analysis of Variance (ANOVA) and for categorical variables using a chi-square test/Fisher's exact test if cell frequency is <5.

### 3.3. The Variations of Tear Film Function at the Check Points between HOCL and Placebo Treatment Groups

#### 3.3.1. Primary Outcomes of Ocular Complaint Scores Slowly Reduced in Both Groups in the following Three Visits (Table 2)

There is no statistical significant differentiation between them in the symptom score reduction of the 15^th^ day (*p* = 0.061) and 30^th^ day (*p* = 0.055) and 55^th^ days (*p* = 0.052); results reveal a 10.57 ± 0.13 (^∗∗^*p* < 0.01) and 12.54 ± 0.17 (^∗∗^*p* < 0.01) decrease in calculated symptom scores after 55 days of atomization management considering the beginnings in both groups, respectively; the difference between them is statistic significant (^∗∗^*p* < 0.01) (Table 2). Results for individual complaint score show that the HOCL brings more benefit than that of control.

#### 3.3.2. Results Show That Both Schirmer's and TBUT Have Been Ameliorated in Both Groups (Table 2)

Tear volume scores (Schirmer's) have increased after the treatment; these increases were 3.27 ± 0.10 and 6.29 ± 0.10 in the contrast and HOCL groups after 55 days of treatment. There are statistical differences between the HOCL and placebo groups in the increased value (Schirmer's) after 55 days of application (^∗∗^*p* < 0.01) (Table 1). The TBUT in the HOCL were always higher than those of the placebo after therapy over a treatment period. After 55 days of management, TBUT were 7.32 ± 1.72 s and 9.22 ± 1.41 s in the placebo and HOCL groups, separately. The alterations between both groups are statistical difference (^∗^*p* < 0.05).

#### 3.3.3. Results Show That Conjunctive Congestion Has Been Alleviated by the Atomization Treatment

The decreased values on the conjunctive congestion of the both groups are 0.23 ± 0.07 and 0.45 ± 0.06 (^∗∗^*p* < 0.01) between the beginning and last interviews, respectively (Table 1). The corneal staining has also been decreased after therapy: decreased figures are 0.42 ± 0.03 and 0.37 ± 0.02 in the placebo contrast and HOCL groups (^∗^*p* < 0.05), respectively. There is a statistical difference in both groups at the final clinical assessment (corneal staining score > 1 at the beginning) (Table 1).

### 3.4. The Inflammation Factors of MMP-9 and IL-2 Represent the Ocular Surface Inflammation Reactions

#### 3.4.1. MMP-9 and IL-2 Biomarker

MMP-9 and IL-2 levels were measured at the beginning and last of the study in the tears' components of the recruiter by the Inflammation Dry® test. 16 subjects out of 27 (59.26%) from the HOCL group and 16 subjects out of 26 (61.54%) from the placebo groups demonstrate MMP-9-positive results in the left eye at the 1^st^ day. Three out of 27 (11.11%) from the HOCL group and 12 out of 26 (46.15%) subjects from the placebo contrast group displayed MMP-9-positive results in the left eye at the 55^th^ day. The MMP-9 shows the inflammation difference between those groups is significant (*Z* = 0.896,  ^∗∗^*p* = 0.002 < 0.01); 15 individuals out of 27 (55.55%) from the HOCL group and 15 out of 26 (57.69%) from the placebo groups manifest IL-2-positive results in the left eye at the 1^st^ day. Two out of 27 (7.41%) from the HOCL group and 10 out of 26 (38.46%) subjects from the placebo contrast group express IL-2-positive results in the left eye at the 55^th^ day. The IL-2 shows the inflammation difference between them is significant (*Z* = 0.659,  ^∗∗^*p* = 0.001 < 0.01).

### 3.5. The *Demodex* Data

The *Demodex* detection and calculation through light microscopy between the first and the final examination.

#### 3.5.1. *Demodex* Count

A total of 27 HOCL treatment patients (54 eyes, 18 females and 9 males, 37.84 ± 1.02 years) and 26 placebo individuals (52 eyes, 17 females and 9 males, 38.30 ± 1.24 years), matched by gender and age, were consecutively recruited for this study. The medium number of *Demodex* mites on three eyelashes per patient at the enrollment check of HOCL group for *Demodex* at the first day is 10, while the last data of HOCL group is 2 (*Z* = −4.642,  ^∗∗^*p* < 0.01). The medium figure of *Demodex* on three cilia per patient at the enrollment check of the placebo group for *Demodex* at the first day was 11, while the last result of placebo group is 6 (*Z* = 2.742, *p* > 0.05). When we analyze the data of both groups, the difference is statistically significant (*Z* = −2.310,  ^∗^*p* = 0.032 < 0.05).

#### 3.5.2. The Survival Time (ST) of *Demodex*

**Figure 2:**
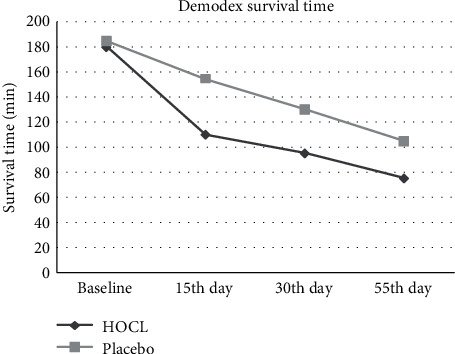
The survival time of *Demodex.*

The mean ST of *Demodex* mite in particular treatments are shown in Figure 2 (survival time of *Demodex*). The average ST at different check points in the HOCL group is 110.75 (108.50 ± 24.50) min, 95.50 (90.25 ± 14.50) min, and 75.25 (73.48 ± 8.50) min which are significantly lower than the average ST at different check points in the placebo group which are 155.50 (160.10 ± 21.50) min, 130.25 (128.25 ± 16.50) min, and 105.75 (102.50 ± 14.50) min (^∗∗^*p* < 0.01).

#### 3.5.3. The *Demodex* Mite Eradication Rate

**Figure 3:**
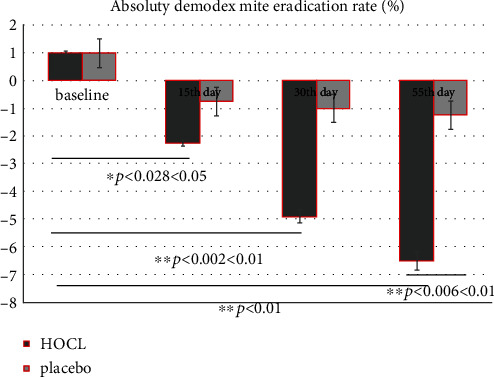
The *Demodex* mite eradication rate.

The *Demodex* counts and ocular parameters at each checkpoint of treatment were also compared (Figure 3 the *Demodex* mite eradication rate). The *Demodex* count in the HOCL group has been reduced by −2.25 ± 0.84 after the 15^th^ day's management; meanwhile, the *Demodex* count in the placebo group has been downregulated by −0.74 ± 0.03 after the 15^th^ day's therapy (*p* = 0.056 > 0.05). There is significant differentiation in the *Demodex* mite eradication rate between them at the 55^th^ day (^∗∗^*p* = 0.006 < 0.01). Compared with that of the placebo, the *Demodex* mite eradication rate of HOCL is statistically significant at each checkpoint (^∗^*p*15^th^ vs. 1^st^ day = 0.028 < 0.05; ^∗∗^*p*30^th^ vs. 1^st^ day = 0.002 < 0.01; ^∗∗^*p*55^th^ vs. 1^st^ day = 0.0018 < 0.01).

## 4. Discussion

### 4.1. The Inflammation of Dry Eye

Dry eye is a chronic recurrent ocular surface disease that most patients' complaints or signs of tear film homeostasis eruption with multiple pathological reactions and the disease or dysfunction of tears fluid producing cells/glands that result in the erratic tear film [35]. Tear eruption is accompanied by raising tear osmotic pressure (local or diffuse area) which induces stress signaling pathways in the ocular surface epithelium and resident immunologic cells and triggers the production of innate inflammatory molecules that arouse the vicious self-perpetuating circulation which leads to being further downregulated in tear film function and worse symptoms. Hyperosmolarity stress has been shown to trigger mitogen-activated protein kinase (MAPK) pathway and stimulate secretion of proinflammatory cytokines (e.g., IL-1*β*, IL-2, IL-6, and TNF-*α*), chemokines, and matrix metalloproteinase such as MMP-3 and MMP-9 as well as cytokine apoptosis [36]. The connection of these inflammatory mediators is intricate which has been shown to affect themselves; the Meibomian gland dysfunction (MGD) “-Relative” dry eye is the leading one, thus amplifying the inflammatory cascade which leads to cornea epithelial barrier disruption, conjunctivae goblet cell loss, and meibomian glandular dysfunction [37]. This manifests the pathological closed loop making the treatment effects uncertain and disease recurrent.

### 4.2. The Natural of HOCL to the Ocular Diseases

Pure HOCl is released as the element of the human immune reaction [32]. During the “oxidative burst,” small, highly reactive molecules, such as free HOCl, are generated as leukocyte responds to the pathogenesis of organisms [38]. This element is an oxidant that kills bacteria through the protein and lipid halogenations and/or per oxidation process [39], which has a diffuse spectrum of activity and exhibits rapid killing vitality [40]. The form of HOCL 0.01% (Avenova®, Nova Bay Pharmaceuticals, Inc., Emeryville, California, USA) is commercially formulated free of sodium hypochlorite with the pH of 3.5-6.0 which has been used in the ocular sickness that has been proved to be safe and effective: Yin et al. and Gold et al. [41, 42] found that 0.01% HOCL significantly reduced inflammation and was effective in killing >99.9% of tested pathogenesis microorganisms without side effect on the ocular surface; HOCL is a potent oxidizing ingredient effective against a wide spectrum of organisms, including the most common bacteria implicated in endophthalmitis after surgery or trauma [9, 32]. Ngo et al. [43] evaluated the comfort levels of several eyelid cleansing products in the treatment of blepharitis associated with *Demodex* folliculorum who have achieved the conclusion that the HOCL 0.01% has the highest degree of patients' comfort.

### 4.3. HOCL Can Alleviate the Inflammation Reaction of Ocular Surface

Ocular inflammations and hyperosmolarity stress are key components of the pathologic circle for the chronic dry eye. Our data show that the ocular inflammation factors between HOCL and placebo group are significantly different (*Z* = 0.896,  ^∗∗^*p* = 0.002 < 0.01; Z = 0.659,  ^∗∗^ *p* = 0.001 < 0.01) at the end of experiment which confirms with the previous study that HOCL can reduce inflammatory factors, such as decreasing the activity of histamine and interleukin-2 (IL-2) and matrix metalloproteinase-9 (MMP-9) (Table 3) all of which are involved in the development of irritation and itching [44]. HOCL has been shown to have effect on controlling the biofilms and wound healing of ocular epithelium [45]. Some authors [43, 46] have reported the decreasing in the figure of *Demodex* mites by HOCL. These biological activities are largely due to the per oxidation reaction property. But the traditional use efficiency of HOCL to the ocular surface is limited for its instability inwardness.

### 4.4. The Mechanism of Ultrasonic Atomization Ocular Surface Device

Ultrasonic atomization moistening device is an extraordinary method for ocular surface which comprises a liquid box and a ventilation high-humidity transparent mirror room around the ocular surface. The atomization system is arranged inside the liquid box, and the atomization system is used for atomizing water in the liquid box. A second gas outlet hole is formed in the liquid box, the first gas inlet hole is formed in the mirror room, and the first gas inlet hole is mixed with the second gas. According to the ultrasonic atomization ocular surface eye moistening device, a high-humidity environment is formed around the ocular surface; the dry eye symptom can be alleviated by the time-depended highly concentrated drugs whose droplets continuously, uniformly, and comprehensively overlay the eyelid, conjunctiva, and cornea and maximize the contact area between the ocular tissue and liquid and there of accelerating drug utilization [47].

### 4.5. The Clinic Tear Film Data of Ocular Surface

A series of clinical researches have shown that the ultrasonic atomization distinguishing using kinds of liquids improves the meibum expression, increases tear fluid, remits the symptoms and signs of DED, and stabilizes the tear film (Table 2) [47–49]. The placebo contrast in this study was 0.1% Purified Sodium Hyaluronate. A public study showed that ultrasonic atomization with saline alone can also ameliorate the symptoms and signs (including Schirmer's I test, TBUT, and corneal fluorescent staining). Compared with the artificial tears (0.1% Purified Sodium Hyaluronate, Santen Pharmaceutical Co. Ltd.), the treatment effect of the placebo ultrasonic atomization group due to Schirmer's is significantly inferior than that of HOCL (^∗∗^*p* < 0.01). The causes for the difference are the chemical nature of HOCL which induces a strong per oxidation and/or halogenation reaction helps for the penetratation ability of eye droplets to the ocular surface. Therefore, in this study, there is statistical difference in the amelioration of TBUT at the endpoint of this trial (^∗^*p* < 0.05) which relates to the significant alleviation effect on the enhancement of oxidizing reactions through the ultrasonic atomization drug deliver system.

### 4.6. The Pathogenesis Mechanism and the Treatments of *Demodex* on the MGD-EDE

The *Demodex* blepharitis has been the hotspot issue by both ophthalmologists and dermatologist in the past decades. Several pathogenesis mechanisms of *Demodex* blepharitis have been elaborated in previous studies. First, *Demodex* mites can block the sebaceous ducts and hair follicles mechanically causing epithelial hyperplasia and keratinization, while debris or waste from *Demodex* mites could diminish innate immune response or the inflammatory reactions. Second, *Demodex* mites damage the habitat at which they live by persistent adjustment and invasions [50].

Since the *Demodex* mite is the pathogenesis for blepharitis and Meibomian gland dysfunction, there are kinds of treatments that have been explored. Different tea tree essential oil (TTO) products are now broadly introduced in *Demodex* blepharitis [51–53]. Liu et al. [50] reported that the okra eyelid dressing effectively eliminated *Demodex* mites both in vivo and in vitro, whose application was associated with the least ocular discomforts. Besides up-to-data drugs, a previous study also show that the eye care practitioners (ECPs) should additionally consider both HOCl-based and TTO cleansers as the first-line choice [53]. Murphy et al. [46] have reported a reduction in the amount of *Demodex* mites with treatment of HOCL which coincides with our study. The treatments for the ocular *Demodex* are such a hot issue that several clinic research protocols are undergoing; the pure HOCL shows amazing prospect.

In this study, the *Demodex* count data of the Table 4 has been decreased from 10 to 2 parasites per cilia at the HOCL group (*Z* = −4.642,  ^∗∗^*p* < 0.01), from 11 to 6 parasites per cilia at the placebo contrast group (*Z* = 2.742, *p* > 0.05) at the endpoint of this clinic trail that the difference between these groups of the variation is significant (*Z* = −2.310,  ^∗^*p* = 0.032 < 0.05) which have seemingly slight decrease when making the determination of the clinical improvements. The exact links between the clinical symptoms and parasite burden remain somehow elusive. Gao et al. [51] have introduced this method of *Demodex* counts with the account of mites recorded in our study, both at the start and at the end of trial. A weekly eyelid scrub with 50% TTO has been verified successful in killing ocular *Demodex* infestation, and counts as low as zero have been proved in a previous study after 4 weeks of treatments [52, 53]. This present study reflects that HOCL has the same effect with that of TTO in terms of *Demodex* eradication on the eyelid tissue; it significantly shortens the survival time of *Demodex* in vivo, the average ST at different check points in the HOCL group is 110.75 (108.50 ± 31.50) min, 95.50 (90.25 ± 14.50) min, and 75.25 (73.48 ± 8.50) min alone which are significantly shorter compared with the average ST in the placebo group which are 155.50 (160.10 ± 21.50) min, 130.25 (128.25 ± 16.50) min, and 105.75 (102.50 ± 14.50) min (^∗∗^*p* < 0.01) (Figure 2). Therefore, HOCL is proved to be a novel treatment for *Demodex* blepharitis by shortening the average survival time.

Additionally, compared with the traditional application of 50% TTO treatment for 3 months in a row, we found that there was no only obvious differentiation between them (*Demodex* mite eradication rate between the two groups at the 55^th^ day) (^∗∗^*p* = 0.006 < 0.01) (Figure 3) with regard to the average *Demodex* figures, but the successful killing rate is higher at different check points of this clinic trail in HOCL groups than that of control (^∗^*p*15^th^ vs. 1^st^ day = 0.028 < 0.05; ^∗∗^*p*30^th^ vs. 1^st^ day = 0.002 < 0.01; ^∗∗^*p*55^th^ vs. 1^st^ day = 0.0018 < 0.01) (Figure 3). The HOCL is a natural immunity reaction of the human body; our data shows no discomforts which coincide with previous study [12, 14, 44, 51]. Otherwise, many discomforts such as allergic reactions, contact dermatitis, and ocular irritations are well-known adverse reactions of TTO proposal [54]. The lower incidence of adverse reactions will ensure higher quality treatment compliance of patient as the reliable choice for MGD-EDE patients.

Due to the limitations of the small quantity of recruit in the present study, the effect of HOCL through ultrasonic atomization for *Demodex* blepharitis/MGD-DED needs further confirmation in a larger cohort and the observation for the alleviation of MGD-DED symptoms needs a long period. The anti-inflammation effects of the pure HOCL in *Demodex* blepharitis are elaborated, the mechanisms of the acaricidal effects of HOCL remain to be fully expound. In order to make sure for the symptom alleviation during the chronic disease management process of dry eye, future studies are required to clarify the tenet of HOCL to cure the *Demodex* blepharitis as well as maintain the ocular surface tear bio film stability.

## 5. Conclusion

We summarized the clinic data that the pure HOCL can improve the eradication rate of the *Demodex* mite by shortening its average survival time.

The HOCL induces lipid per oxidation reaction to the ocular surface pathogenesis microorganisms that the ocular surface inflammation can be alleviated.

The ultrasonic atomization drug delivery system helps for the drug usage.

## Figures and Tables

**Table 1:** Characteristic of the patients at baseline.

Variables	Hypochlorous acid (HOCL, *N* = 27)	Placebo (*N* = 26)	Statistical	*p* value
Demographics				
Age (year)				
Mean ± SEM	37.84 ± 1.02	38.30 ± 1.24	*Z* = 0.1921	0.8343
Sex				
Male, *n* (%)	9 (32.29%)	9 (34.61%)		
Female, *n* (%)	18 (67.71%)	17 (65.38%)	*χ* ^2^ = 0.0583	0.6743
Best corrected visual acuity				
Mean ± SEM	0.82 ± 0.04	0.83 ± 0.02	*Z* = −0.6572	0.5692
Eye symptom score				
Mean ± SEM	13.38 ± 0.32	14.17 ± 0.42	*Z* = 0.2192	0.6172
Meibum quality score				
0, *n* (%)	11 (40.83)	10 (38.46)	*Z* = 0.4412	0.5658
1, *n* (%)	9 (35.33)	8 (30.77)		
2, *n* (%)	5 (21.32)	6 (23.08)		
3, *n* (%)	2 (6.67)	2 (7.69)		
Eyelid edge change score				
Mean ± SEM	1.58 ± 0.06	1.71 ± 0.06	*Z* = −0.0921	0.4835
Meibum expression score				
Mean ± SEM	2.43 ± 0.18	2.65 ± 0.2	*Z* = −0.7112	0.5162
Conjunctive congestion score				
0, *n* (%)	7 (24.50)	6 (23.08)	*Z* = 0.1031	0.8917
1, *n* (%)	12 (48.00)	11 (42.31)		
2, *n* (%)	4 (14.81)	7 (17.28)		
3, *n* (%)	4 (14.81)	2 (7.69)		
Corneal labeling score				
Mean ± SEM	0.58 ± 0.07	0.60 ± 0.07	*Z* = −0.4231	0.7325
Schirmer's I test				
Mean ± SEM	5.57 ± 0.31	5.62 ± 0.18	*Z* = −0.0347	0.4372
None interfere tear breakup time(s)				
Mean ± SEM	4.23 ± 0.18	4.08 ± 0.20	*Z* = 1.128	0.2327

**Table 2:** Summary of efficacy endpoints between hypochlorous acid (HOCL) and placebo groups.

Visit duration	HOCL (*n* = 27), mean ± SEM	Placebo (*n* = 26), mean ± SEM	*p* value by ANOVA for HOCL vs. placebo
(a) Ocular surface disease index (OSDI) scores			
1^st^ day	18.65 ± 5.20	21.54 ± 4.98	0.058
15^th^ day	14.25 ± 4.45	13.68 ± 5.02	0.061
30^th^ day	11.14 ± 4.14	10.89 ± 4.98	0.055
55^th^ day	8.08 ± 4.32	9.00 ± 6.68	0.052
(b) Schirmer's test average of both eyes			
1^st^ day	7.09 ± 2.34	6.65 ± 1.82	0.411
15^th^ day	9.22 ± 2.12	7.64 ± 2.04	0.053
30^th^ day	12.28 ± 3.28	9.90 ± 4.21	<0.05^∗^
55^th^ day	13.38 ± 2.73	9.92 ± 2.12	<0.01^∗∗^
(c) Tear film break-up time (TBUT) average of both eyes			
1^st^ day	4.78 ± 1.12	4.86 ± 1.02	0.423
15^th^ day	4.89 ± 1.56	4.97 ± 1.25	0.351
30^th^ day	6.13 ± 1.13	6.09 ± 1.08	0.459
55^th^ day	9.22 ± 1.41	7.32 ± 1.72	<0.05^∗^
(d) Conjunctivae labeling average of both eyes			
1^st^ day	0.86 ± 0.19	0.88 ± 0.20	0.677
55^th^ day	0.41 ± 0.27	0.65 ± 0.25	<0.01^∗∗^
(e) Corneal labeling average of both eyes			
1^st^ day	1.05 ± 0.09	1.24 ± 0.12	0.406
55^th^ day	0.68 ± 0.11	0.82 ± 0.09	<0.05^∗^

**Table 3:** Figures of subjects positive for inflammation biomarker between hypochlorous acid and placebo groups.

Biomarker variation	HOCL (*N* = 27), negative positive		Placebo (*N* = 26), negative positive		*p* value for HOCL vs. placebo *Z*-test
	*N* (%)	*n* (%)	*n* (%)	*n* (%)	
MMP-9					
1^st^ day	11 (40.74)	16 (59.26)	10 (38.46)	16 (61.54)	
55^th^ day	24 (88.88)	3 (11.11)	14 (53.84)	12 (46.15)	*Z* = 0.896, ^∗∗^*p* = 0.002
IL-2					
1^st^ day	12 (44.44)	15 (55.55)	11 (42.31)	15 (57.69)	
55^th^ day	25 (92.59)	2 (7.41)	16 (61.54)	10 (38.46)	*Z* = 0.659, ^∗∗^*p* = 0.001

*N*: number of subjects in designated treatment; *n*: number of subjects in special category; %n (number of subjects in special category)/N (number of subjects in designated treatment) × 100; ^∗^*p* < 0.05 and^∗∗^*p* < 0.01.

**Table 4:** The *Demodex* count between HOCL and placebo groups [P50 (P25, P75)].

Groups	Cases	1^st^ day	55^th^ day	*Z*	*p*
HOCL	54	10 (7,15)	2 (0,4)	-4.642	<0.01
Placebo	52	11 (8,18)	6 (4,14)	2.742	>0.05
*Z*		-0.892	-2.310		
*p*		0.431	0.032<0.05		

## Data Availability

The data sets used and/or analyzed during the present study are available from the corresponding author on reasonable request.
